# Genetic Pattern and Demographic History of *Salminus brasiliensis*: Population Expansion in the Pantanal Region during the Pleistocene

**DOI:** 10.3389/fgene.2018.00001

**Published:** 2018-01-17

**Authors:** Lívia A. de Carvalho Mondin, Carolina B. Machado, Emiko K. de Resende, Debora K. S. Marques, Pedro M. Galetti

**Affiliations:** ^1^Departamento de Ciências Biológicas, Universidade do Estado de Mato Grosso, Tangará da Serra, Brazil; ^2^Laboratório de Biodiversidade Molecular e Conservação, Departamento de Genética e Evolução, Universidade Federal de São Carlos, São Carlos, Brazil; ^3^Embrapa Pantanal, Empresa Brasileira de Pesquisa Agropecuária, Corumbá, Brazil

**Keywords:** approximate Bayesian computation, mitochondrial markers, interglacial period, neotropical fish, Upper Paraguay basin

## Abstract

Pleistocene climate changes were major historical events that impacted South American biodiversity. Although the effects of such changes are well-documented for several biomes, it is poorly known how these climate shifts affected the biodiversity of the Pantanal floodplain. Fish are one of the most diverse groups in the Pantanal floodplains and can be taken as a suitable biological model for reconstructing paleoenvironmental scenarios. To identify the effects of Pleistocene climate changes on Pantanal’s ichthyofauna, we used genetic data from multiple populations of a top-predator long-distance migratory fish, *Salminus brasiliensis*. We specifically investigated whether Pleistocene climate changes affected the demography of this species. If this was the case, we expected to find changes in population size over time. Thus, we assessed the genetic diversity of *S*. *brasiliensis* to trace the demographic history of nine populations from the Upper Paraguay basin, which includes the Pantanal floodplain, that form a single genetic group, employing approximate Bayesian computation (ABC) to test five scenarios: constant population, old expansion, old decline, old bottleneck following by recent expansion, and old expansion following by recent decline. Based on two mitochondrial DNA markers, our inferences from ABC analysis, the results of Bayesian skyline plot, the implications of star-like networks, and the patterns of genetic diversity (high haplotype diversity and low-to-moderate nucleotide diversity) indicated a sudden population expansion. ABC allowed us to make strong quantitative inferences about the demographic history of *S*. *brasiliensis*. We estimated a small ancestral population size that underwent a drastic fivefold expansion, probably associated with the colonization of newly formed habitats. The estimated time of this expansion was consistent with a humid and warm phase as inferred by speleothem growth phases and travertine records during Pleistocene interglacial periods. The strong concordance between our genetic inferences and this historical data could represent the first genetic record of a humid and warm phase in the Pantanal in the period since the Last Interglacial to 40 ka.

## Introduction

The genetic diversity and population structure of species are largely determined by intrinsic traits, contemporary factors that include anthropogenic activities and historical events that have affected species over geological time (Avise, 2000; Osborne et al., 2014). Phylogeographic studies have shown that the genetic diversity patterns of several populations were shaped by past geological and climatic events (Hewitt, 1996; Bonaccorso et al., 2006; Carnaval et al., 2009; Thomaz et al., 2015; Cabanne et al., 2016).

The Pleistocene (2.58–0.01 million years ago) was marked by worldwide climatic changes involving multiple successive cycles of glacial and interglacial events, associated with abrupt and dramatic changes in temperature and precipitation (Webb and Bartlein, 1992). Overall, the environmental conditions varied from warm and wet during the interglacial to cold and dry during the glacial periods (EPICA Community Members, 2004).

Although there are no records of large ice sheets covering the landscape, as happened in the Northern Hemisphere, climatic changes during the Pleistocene also drastically affected Southern Hemispheric biomes (Vuilleumier, 1971; Hewitt, 1996). Several studies have reported contractions and range expansions in the Amazon and the Atlantic Forest (Harris and Mix, 1999; Carnaval et al., 2009; Ribas et al., 2012; Cabanne et al., 2016; Ledo and Colli, 2017), replacement by savannas and arid lands (Wang et al., 2004; Carnaval and Bates, 2007; Thomé et al., 2016), climatic disturbances in humid and arid areas in the Andes (Bräuning, 2009), and fluctuations in sea levels affecting the Atlantic coast (Thomaz et al., 2015; Ponce and Rabassa, 2016). The magnitude of climatic oscillations varied in different parts of South America. However, all of these events impacted the biodiversity, driving extinction episodes, speciation, intraspecific divergence, and demographic oscillations (Vuilleumier, 1971; Hewitt, 1996; Beheregaray, 2008). However, it remains poorly understood how these historical events affected the Pantanal biome and its biodiversity.

The Pantanal is a large seasonal floodplain located in the center of South America. This region is entirely contained in the Upper Paraguay basin, which is a complex drainage network due to its geological history (Assine, 2015). Pantanal was formed recently and has mainly been associated with the Andean uplift (Assine, 2015). However, geomorphological (Assine and Soares, 2004) and palynological (Ferraz-Vicentini and Salgado-Labouriau, 1996) data have shown that the paleoclimatic fluctuations have promoted considerable landscape changes since the Late Pleistocene. Although there are historical records of environmental changes, little is known of the effects of these changes on biodiversity in the Pantanal (Márquez et al., 2006; Lopes I.F. et al., 2007; Santos et al., 2008).

Fish are one of the most diverse groups in the Pantanal floodplains (Alho, 2008). Because their distribution is restricted to freshwater drainages, which in turn reflect geomorphological or climatic changes, these animals are a suitable biological model for reconstructing paleoenvironmental scenarios (Montoya-Burgos, 2003). To try to identify any effects of Pleistocene climate changes in fish, we used genetic data from multiple sites of *Salminus brasiliensis*. As a top predator distributed throughout the La Plata basin (Lima and Britski, 2007), this species represents an important resource in the Pantanal floodplains. Although it plays an important ecological role in controlling the structure of the ecosystem, the genetic diversity of *S*. *brasiliensis* in the Upper Paraguay basin is unknown. Assessing the population genetic pattern of the existing species can identify how its genetic variation was affected by past climate changes and can also provide insights into its demographic response (Pil et al., 2017).

Here, we focused on assessing the genetic diversity and pattern of *S*. *brasiliensis* sampled in nine rivers to trace its demographic history in the Upper Paraguay basin using a statistical framework to test the following five scenarios: constant population (null hypothesis), old expansion, old decline, old bottleneck followed by recent expansion, and old expansion followed by recent decline. We specifically investigated whether Pleistocene climate changes affected past *S*. *brasiliensis* demography. If so, we expected to find a population reduction signature during glacial periods due to climatic conditions that reduced the habitat, when the Pantanal region was almost dry. Further, a population expansion signature was expected in the interglacial periods, when the wetlands were re-established, increasing fluvial discharge and suitable habitats for the biological model. After reconstructing the most probable demographic history for *S*. *brasiliensis* based on two mitochondrial markers (CytB and Dloop), we associated changes in population size over time with known regional climatic fluctuations. Our findings might contribute to the conservation and management of natural populations of *S*. *brasiliensis* from the Upper Paraguay basin by being used to predict whether these populations will be able to persist in future scenarios of environmental change.

## Materials and Methods

### Ethics Statement, Sampling Collection, DNA Extraction, and Amplification

**FIGURE 1:**
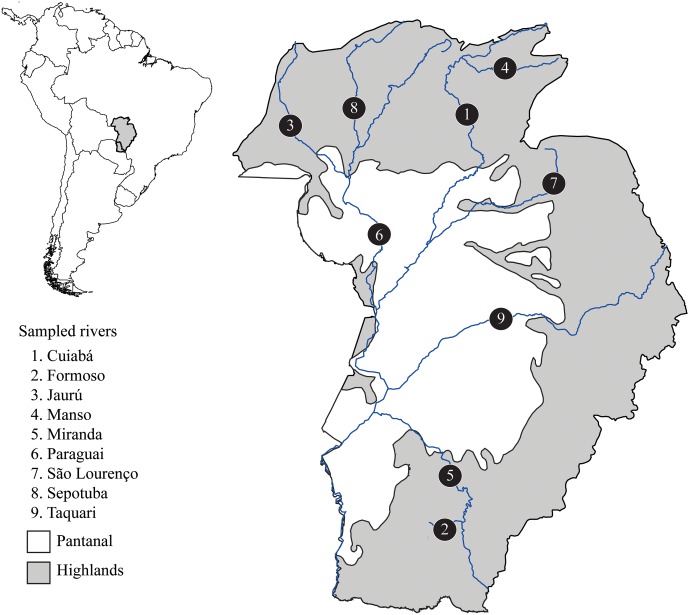
Map of the Upper Paraguay basin in Brazilian territory indicating rivers where *Salminus brasiliensis* were sampled. Populations are labeled with numbers. The Pantanal area is in white and represents the lowlands (below 60 m above sea level).

This study was developed in accordance with Brazilian law, approved by the ethic committee on animal use at Federal University of São Carlos (CEUA number 5765010416), and carried out under a temporary scientific collection license number 32217-4 provided by ICMBio-SISBIO. A total of 52 *S*. *brasiliensis* individuals were sampled in nine rivers from the Upper Paraguay basin (**Figure 1** and Supplementary Table S1). We sampled a small fragment of the caudal fin and then returned the fish to the river. All tissue samples were preserved in absolute ethanol and stored at -20°C. Total genomic DNA was extracted using the saline precipitation protocol (Aljanabi and Martinez, 1997).

We amplified partial sequences of two mitochondrial DNA markers: Cytochrome B (CytB) and Dloop (see Supplementary Table S2 for primers and references). Polymerase Chain Reactions for both markers (PCR) occurred in 25 μL of final volume containing 16.9 μL of mili-Q water, 2.5 μL of 10x Invitrogen PCR buffer (1x), 2.5 μL of dNTPs mix (0.25 mM), 1 μL of MgCl_2_ (2 mM), 0.5 μL of each primer (0.2 mM), 0.1 μL of Platinum^®^Taq Polymerase (0.5 unit) and 1 μL of DNA template (50 ng/μL). The annealing temperature of PCR thermal profile was described in Supplementary Table S2. All PCR products were purified using the 20% Polyethylene glycol protocol (Lis and Schleif, 1975). Fragments were sequenced using ABI3730XL automatic sequencer. All the sequences were aligned automatically using the multiple alignment ClustalW and minor manual adjustments were made to improve it in BioEdit (Hall, 1999). The GenBank accession numbers are given in Supplementary Table S1.

### Genetic Diversity and Population Structure

The genetic diversity indices for each population, including number of haplotypes, haplotype diversity, and nucleotide diversity, were calculated in DnaSP 5.0 (Librado and Rozas, 2009). We also reconstructed the haplotype networks for each molecular marker using the median-joining method (Bandelt et al., 1999) included in PopART (Population Analysis with Reticulate Trees – Leigh and Bryant, 2015).

We investigated whether there were distinct mitochondrial genetic clusters by assigning individuals to populations using a Bayesian model-based approach implemented in Bayesian Analysis of Population Structure (BAPS) v. 6.0 (Corander et al., 2008). BAPS identifies clusters based on nucleotide frequencies in DNA sequences. We concatenated mitochondrial markers and conducted a mixing analysis without spatialization (option chosen “clustering of group of individuals”). This method determines which combination of predetermined samples is best supported by the data. The program was run for 10 replicates for each K (1–10). The best clustering partition was determined by the highest value of likelihood and highest probability determined by the program.

### Inference and Hypotheses Tests of Demographic Histories

Because no genetic population structuring was found in *S*. *brasiliensis* using BAPS (Supplementary Figure S1 and Supplementary Table S3), we drawn all scenarios based on historical demographic changes in a single population. Demographic history was inferred through a Bayesian skyline plot (BSP) analysis (Drummond et al., 2005) implemented in BEAST 2.4.4 (Bouckaert et al., 2014). BSP assumes a single panmictic population and uses inferred patterns of coalescence to fit a demographic model to a set of sequence data (Drummond et al., 2005).

First, we determined the best evolutionary model for each molecular marker in JModelTest 2.1.4 (Posada, 2008) using the Bayesian information criterion. The CytB and Dloop markers were set to evolve according to a HKY and HKY+I models, respectively. In BEAST 2.4.4, we chose as the tree prior the coalescent Bayesian skyline with both linked mitochondrial DNA markers to perform the BSP analysis. Priors used in this analysis were kept in default. To date the past demographic events, a molecular clock was calibrated using the mutation rate defined for the *Salminus* genus (Machado et al., unpublished), using the relaxed lognormal model: 6.31 × 10^-3^ (CytB) and 1.935 × 10^-2^ (Dloop) mutations per site per million years. This rate was based on a previous topology calibrated from the genus rooted with *Brycon* specimens (Machado et al., unpublished). This topology was reconstructed using two mitochondrial and two nuclear markers and calibrated using a biogeographic event, the uplift of the Eastern Andes Cordillera (Hoorn et al., 1995), which separated the Magdalena and Amazon paleobasins and is consistent with the divergence of *S. affinis* from all other *Salminus*.

Three independent Markov chains were initiated from random trees, run separately for 10 million generations, and sampled at intervals of 10000 steps, 25% of which were discarded as burn-in. The log and tree files for each independent Markov chain were combined using LogCombiner in BEAST 2.4.4. Tracer v1.5 (Rambaut et al., 2014) was used to check the convergence of the runs according to the effective sampling size (>200) and reconstruct the population dynamic over time through the Bayesian Skyline reconstruction option.

**FIGURE 2:**
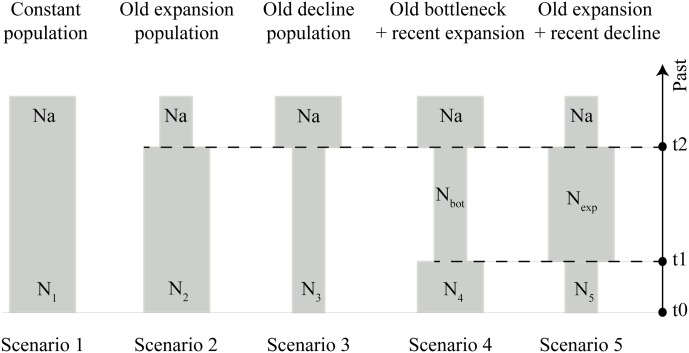
Schematic representation of five demographic scenarios, including model parameters of *S. brasiliensis*, tested by approximate Bayesian computation (ABC). Time and effective population size are not strictly to scale.

To corroborate the findings on the population expansion of *S*. *brasiliensis* in the Upper Paraguay basin suggested by the BSP analysis, we tested five demographic scenarios that could have happened during the Late Pleistocene (expansions and bottlenecks) using an approximate Bayesian computation (ABC) framework in DIYABC v2.1.0 (Cornuet et al., 2014): constant population (null hypothesis), old expansion, old decline, old bottleneck followed by expansion, and old expansion followed by decline (**Figure 2**). ABC approach allows a quantitative evaluation of the demographic and evolutionary history by strictly contrasting realistic models defined *a priori* and estimating relevant parameters (Beaumont et al., 2002).

All scenarios assumed an initial ancestral population (Na), demographic changes occurring during the Late Pleistocene (2–120 ka for t1, and 4–120 ka for t2), and recent populations represented in t0 (**Figure 2**). The prior distribution of demographic parameters and the mutation rate are listed in Supplementary Table S4. For each tested scenario, we calculated the following summary statistics: the number of haplotypes, number of segregating sites, variance of pairwise differences, and variance of numbers of the rarest nucleotides at segregating sites. The summary statistics were used for comparison between simulated and observed datasets.

We ran one million simulations for each scenario and used summary statistics and principal component analysis to confirm the good fit of all scenarios with the observed data (Supplementary Figure S2). Then we compared the competing scenarios by estimating their posterior probabilities using polychotomous logistic regression on 1% of simulated datasets closest to the observed data (Cornuet et al., 2008). The best scenario was the one that showed the highest posterior probability with a 95% confidence interval (CI) non-overlapping with other scenarios’ posterior probabilities. In order to evaluate confidence in each scenario, we also calculated the posterior predictive error. Subsequently, we estimated the posterior probabilities of each parameter under the best scenario using a local linear regression on 1% closest simulated data sets and applying a logit transformation to parameter values as suggested by Cornuet et al. (2008). We assessed their precision of parameter estimation through the relative median of the absolute error (RMAE) based on 500 pseudo-observed datasets (pods) under the best scenario. Low RMAE values indicated that the parameter estimated is trustworthy (Cornuet et al., 2008). After these steps, we performed a model verification by evaluating the goodness-of-fit of the best scenario with respect to empirical dataset. This was carried out by simulating 1000 pods under the best scenario. In this step, we used different summary statistics to avoid over-estimating the fit by using the same statistics twice (Cornuet et al., 2010). We selected mean of pairwise differences, Tajima’s D, private segregating sites and mean of number of the rarest nucleotide at segregating sites.

## Results

Fragments of 710 base pairs (bp) of CytB and 571 bp of Dloop were obtained from 52 individuals of *S*. *brasiliensis* from the Upper Paraguay basin. CytB had 20 polymorphic sites, of which 9 were parsimoniously informative, while Dloop had 37 polymorphic sites, of which 26 were parsimoniously informative. No insertions, deletions, or stop codons (for CytB sequences) were observed for either marker.

We identified 21 and 33 haplotypes for CytB and Dloop, respectively (Supplementary Table S5 and Supplementary Figure S1). No common haplotype was shared among all populations in any molecular markers, but a high number of private haplotypes (61.9% for CytB and 66.7% for Dloop) was observed. The distribution of the haplotypes in the network indicated no population structuring in the rivers sampled (Supplementary Figure S1). The haplotype and nucleotide diversity values for each population are shown in Supplementary Table S5. Overall, despite the nucleotide diversity exhibited was low (0.00365 for CytB) to moderate (0.01434 for Dloop), the level of haplotype diversity was high (0.928 for CytB, and 0.975 for Dloop).

**FIGURE 3:**
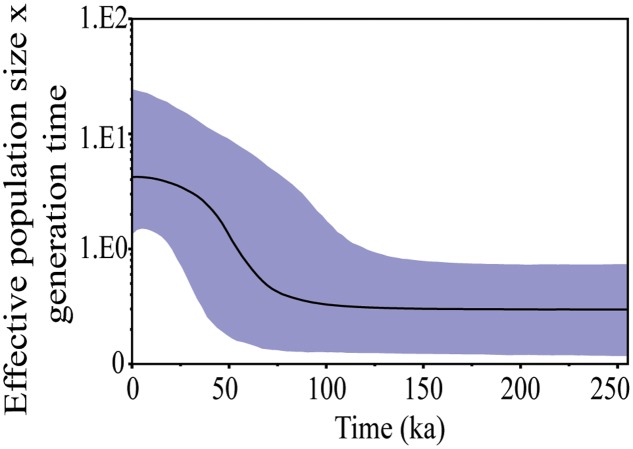
Bayesian skyline plot (BSP) showing change in effective population size of *S*. *brasiliensis* from the Upper Paraguay basin over time based on CytB and Dloop markers. The y-axis, population size × generation time^∗^; x-axis, time (indicated in thousand of years ago). ^∗^Generation time measured in million years.

The BAPS approach clustered all individuals into only one mitochondrial genetic group (Supplementary Figure S1) and the BSP analysis gave the effective population size, which underwent a process of expansion that started approximately 75 ka (**Figure 3**). The greatest expansion was underway through around 40 ka and continued gradually until 20 ka, when the effective population size became stable.

Approximate Bayesian computation analysis also corroborated the old expansion scenario as the most probable one to explain the current genetic diversity of *S*. *brasiliensis* from the Upper Paraguay basin (Supplementary Figure S3). The best scenario showed a posterior probability (PP) of 0.5466 (95% CI: 0.5341–0.5590), while the second PP was observed for scenario 4 (old bottleneck followed by expansion), with 0.2589 (95% CI: 0.2466–0.2712) (Supplementary Table S6). The posterior error rate was 0.303. Features such as the non-overlapping 95% CI among the scenarios’ PPs, and the signal expansion found by BSP analysis and genetic pattern (see section “Discussion”) suggest a greater confidence that scenario 2 (old population expansion) is the best of the proposed scenarios.

The best scenario recovered the ancestral population of *S*. *brasiliensis* (Na = 161000) expanding at 31000 generations ago (i.e., about 62 ka). At present, the median effective population size is estimated at N_2_ = 811000 (**Table 1**). Large confidence intervals are common in such demographic inferences. All our RMAE values are <0.2, except by current effective population size (RMAE = 0.299), indicating the parameters estimated by ABC analysis were reliable values. This was expected because mitochondrial is reliable to detect past demographic parameter estimates (Na and t2) (Cornuet et al., 2010). When we applied the model checking option to the best scenario, we observed on each PCA plane a wide cloud of data sets simulated from the prior, with the observed dataset in the middle of a small cluster of datasets from the posterior predictive distribution (Supplementary Figure S4). This indicate that the scenario 2 fits well the empirical data.

**Table 1:** Parameter estimates generated from a local linear regression on simulated datasets generated from the best scenario.

Parameter	Median	95% Confidence interval	RMAE
N_2_ (Present population)	811000	495000–982000	0.299
Na (Ancestral population)	161000	41500–539000	0.194
t2 (Expansion time)	62 (ka)	9.860–116.6 (ka)	0.093

*The precision of parameter estimations was assessed by computing the relative median of absolute error (RMAE). ka, 1000 years before present.*

## Discussion

High haplotype of genetic diversity and no spatial genetic structuring among populations of *S*. *brasiliensis* were here observed. The results also revealed that its current genetic diversity was strongly influenced by an expansion event that happened during the Pleistocene, likely associated with warm and humid weather conditions during interglacial periods. All of these findings are discussed in detail below.

### Genetic Diversity and Structure of *S*. *brasiliensis*

Previous studies in different hydrographic systems and using other molecular markers, such as RAPD and microsatellites, have determined a great genetic variability for *S*. *brasiliensis* (Ramella et al., 2006; Lopes C.M. et al., 2007; Gomes et al., 2013; Ribeiro et al., 2016; Ribolli et al., 2017). High levels of haplotype diversity seem to be common in migratory freshwater fishes with large populations that occupy heterogeneous environments (see Oliveira et al., 2009). A large effective population size and high migration rates may minimize the effects of genetic drift as a force that decreases intraspecific genetic diversity (Allendorf and Luikart, 2007). Furthermore, the pattern of genetic diversity of *S*. *brasiliensis* also can be attributed to its demographic history. According to Grant and Bowen (1998), high haplotype diversity and low nucleotide diversity is associated with an expansion event after a period of small effective population size. Thus, this process could have introduced and maintained new mutations in the population of *S*. *brasiliensis*, increasing its genetic variability.

No mtDNA genetic structuring was detected in *S*. *brasiliensis*. Its higher migratory capacity (it can move 1000 km during the reproductive season; Petrere, 1985) probably played an important role in causing the lack of spatial genetic differentiation between populations. It is expected that long-distance migratory fishes within a hydrographic basin such as the Upper Paraguay, without physical, abiotic, and/or biotic barriers, will not show a spatial genetic structuring (Pil et al., 2017). However, it seems that *S*. *brasiliensis* has undergone a different process of population differentiation. Ribolli et al. (2017) identified distinct temporal genetic populations of *S*. *brasiliensis* from the Upper Uruguay basin during the reproductive season. According to those authors, individuals or populations of *S*. *brasiliensis* that occupy the same geographic location might be adapted to spawning at different times during the same reproductive season (Ribolli et al., 2017). Because our sample lacks more precise temporal information for some sampled individuals, no time-related genetic population structuring can be inferred here.

### Revisiting the Pleistocene in the Pantanal Region

The demographic history of *S*. *brasiliensis* populations in the Upper Paraguay basin was addressed for the first time in this study, and it was found that a demographic expansion event probably occurred in its early evolutionary history. This took place in the late Pleistocene, before the Last Glacial Maximum (LGM, 23–19 ka). Our inference from ABC analysis, the results of BSP, the implications of star-like networks, and the patterns of genetic diversity (high haplotype diversity and low to moderate nucleotide diversity) indicated a sudden population expansion.

The ABC approach was an efficient tool for reconstructing and testing alternative demographic scenarios; this allowed us to determine the most plausible evolutionary history of *S*. *brasiliensis*, beyond making strong quantitative inferences about its demographic history. We estimated a small ancestral population size that underwent a drastic fivefold expansion, probably associated with the colonization of newly formed habitats. The time estimated for this expansion was consistent with the humid and warm phase inferred from speleothem growth phases and travertine records during Pleistocene interglacial periods (Wang et al., 2004).

Pleistocene climatic oscillations in South America drastically altered freshwater systems by changing hydrological flow and lake distributions as well as rerouting rivers (Stevaux, 2000; Cross et al., 2001; Fritz et al., 2004). As an alluvial plain dominated by rivers, the Pantanal region reveals geomorphological relicts overprinted on its modern landscape that indicate the occurrence of intense historical hydrological rearrangement (Assine and Soares, 2004). Paleochannels, changes in the levels of marginal lakes, and discontinuous sedimentation caused by intermittent flows are examples of relict landforms, which reflect environmental and climatic conditions different from those currently observed (Assine, 2015). Radiocarbon dating and palynological data support the conclusion that during colder and arid events in the Late Pleistocene/Holocene, the Pantanal experienced desert-like conditions with sparse dry vegetation, intermittent flow along the alluvial fan, and the development of paleochannels and erosional surfaces. Further, humid and warm conditions would have influenced the development of lakes and river systems and consequently expanded freshwater surface area (Ferraz-Vicentini and Salgado-Labouriau, 1996; Assine and Soares, 2004; Assine, 2015).

The pulses of the reduction and expansion of the landscape left a phylogenetic signature in regional fauna, leading to a population expansion in bird (Lopes I.F. et al., 2007; Santos et al., 2008) and mammal species (Márquez et al., 2006) stemming from the humid cycles of the Pleistocene. The signature population expansion in *S*. *brasiliensis* was dated to 62 ka. Although there is a lack of regional paleoenvironmental data extending further than 40 ka, speleothem data from the Brazilian Northeast region reveal a pluvial maxima phase at about 61.2–59.1 ka (Wang et al., 2004), reinforcing our findings from *S*. *brasiliensis*. The strong concordance between our genetic inferences and this historical data could represent the first genetic record of a humid and warm phase in the Pantanal in the period since the Last Interglacial to 40 ka.

## Conclusion

In summary, we focused to identify the effect of Pleistocene climatic changes on demographic history of *S. brasiliensis* from the Upper Paraguay basin. Our findings suggest that Pleistocene climate fluctuations fundamentally shaped the genetic diversity and pattern of this species in this region. Coalescent-based analyses, particularly the statistical framework provided by the ABC method, supported an ancient expansion event before the LGM as the best scenario to explain the current genetic diversity of *S*. *brasiliensis* based on two mitochondrial markers.

Understanding how historical events impact genetic diversity is important for predicting whether populations will persist under future environmental changes (Pauls et al., 2013). High genetic variation in populations is desirable, because it provides the basis for evolutionary change in a species, thereby improving the chances of survival in a dynamic environment (Frankham, 1995). Although populations of *S*. *brasiliensis* from the study area show high levels of genetic diversity, our concern over its conservation status cannot wane, because its genetic pool is unique and completely different from the *S*. *brasiliensis* found in the Upper Paraná basin (Machado et al., 2017). We suggest that conservation management of this species should concentrate on maximizing the retention of diversity, and preventing severe population decline due to anthropogenic influence.

## Author Contributions

LCM, CM, ER, DM, and PG conceived the ideas and designed the experiments. LCM, ER, and DM collected the samples. CM and PG performed the bioinformatics analyses. LCM, CM, and PG led the writing, with assistance from ER and DM. All authors read and approved the final manuscript.

## Conflict of Interest Statement

The authors declare that the research was conducted in the absence of any commercial or financial relationships that could be construed as a potential conflict of interest.
