# Supplementary material for: Genetic Pattern and Demographic History of Salminus brasiliensis: Population Expansion in the Pantanal Region during the Pleistocene
Source: Front Genet. 2018 Jan 17;9:1. doi: 10.3389/fgene.2018.00001 (PMC5776086; doi:10.3389/fgene.2018.00001)
Supplement: Supplementary file 1 [file Table_1.DOC]

Table S1: Sample information: ID sample; Locality; Genbank accession number.

| ID sample | River | GenBank Number | |
| --- | --- | --- | --- |
|  |  | CytB | Dloop |
| 131_CUI | Cuiabá | MF683903 | MF683855 |
| 163_CUI | Cuiabá | MF683905 | MF683857 |
| 183_CUI | Cuiabá | MF683906 | MF683858 |
| SB057 | Cuiabá | MF683924 | MF683876 |
| SB059 | Cuiabá | MF683925 | MF683877 |
| SB060 | Cuiabá | MF683926 | MF683878 |
| SB066 | Cuiabá | MF683927 | MF683879 |
| SB069 | Cuiabá | MF463807 | MF463889 |
| SB156 | Cuiabá | MF683931 | MF683883 |
| SB164 | Cuiabá | MF683932 | MF683884 |
| 148_MIR | Miranda | MF683904 | MF683856 |
| SB040 | Miranda | MF683919 | MF683871 |
| SB042 | Miranda | MF683920 | MF683872 |
| SB045 | Miranda | MF683921 | MF683873 |
| SB046 | Miranda | MF683922 | MF683874 |
| SB016 | Miranda | MF463806 | MF463888 |
| 55_SLO | São Lourenço | MF683907 | MF683859 |
| SB033 | São Lourenço | MF683914 | MF683866 |
| SB034 | São Lourenço | MF683915 | MF683867 |
| SB035 | São Lourenço | MF683916 | MF683868 |
| SB037 | São Lourenço | MF683917 | MF683869 |
| SB038 | São Lourenço | MF683918 | MF683870 |
| 65_FOR | Formoso | MF683908 | MF683860 |
| SB010 | Formoso | MF683909 | MF683861 |
| SB011 | Formoso | MF683910 | MF683862 |
| SB048 | Formoso | MF683923 | MF683875 |
| SB152 | Formoso | MF683929 | MF683881 |
| SB013 | Taquari | MF683911 | MF683863 |
| SB023 | Taquari | MF683912 | MF683864 |
| SB025 | Taquari | MF683913 | MF683865 |
| SB147 | Taquari | MF683928 | MF683880 |
| SB153 | Taquari | MF683930 | MF683882 |
| SB174 | Jaurú | MF463808 | MF46389 |
| SB175 | Jaurú | MF683933 | MF683885 |
| SB181 | Manso | MF683934 | MF683886 |
| SB182 | Sepotuba | MF683935 | MF683887 |
| SB183 | Sepotuba | MF683936 | MF683888 |
| SB184 | Sepotuba | MF683937 | MF683889 |
| SB186 | Sepotuba | MF683938 | MF683890 |
| SB188 | Sepotuba | MF683939 | MF683891 |
| SB193 | Paraguai | MF683940 | MF683892 |
| SB194 | Paraguai | MF683941 | MF683893 |
| SB195 | Paraguai | MF683942 | MF683894 |
| SB199 | Paraguai | MF683943 | MF683895 |
| SB207 | Paraguai | MF683944 | MF683896 |
| SB208 | Paraguai | MF463809 | MF463891 |
| SB210 | Paraguai | MF683945 | MF683897 |
| SB211 | Paraguai | MF683946 | MF683898 |
| SB212 | Paraguai | MF683947 | MF683899 |
| SB213 | Paraguai | MF683948 | MF683900 |
| SB214 | Paraguai | MF683949 | MF683901 |
| SB216 | Paraguai | MF683950 | MF683902 |
