# Supplementary material for: Genetic Pattern and Demographic History of Salminus brasiliensis: Population Expansion in the Pantanal Region during the Pleistocene
Source: Front Genet. 2018 Jan 17;9:1. doi: 10.3389/fgene.2018.00001 (PMC5776086; doi:10.3389/fgene.2018.00001)
Supplement: Supplementary file 2 [file Table_2.DOC]

Table S2: Primer information used in this study.

| Marker | Primer name | Primer sequence | Reference | Annealing temperature |
| --- | --- | --- | --- | --- |
| CytB | L14841  H15915 | 5’- AAAAAGCTTCCATCCAACATCTCAGCATGAAA - 3’  5’- AACTGCCAGTCATCTCCGGTTTACAAGAC - 3’ | Irwin et al., 1991  Kocher et al., 1989 | 51^o^C |
| Dloop | DloopL  H16498 | 5’- AGAGCGTCGGTCTTGTAAACC - 3’  5’- CCTGAAGTAGGAACCAGATG - 3’ | Cronin et al., 1993  Meyer et al., 1990 | 54^o^C |

References

Cronin, M. A., Spearman, W. J., Wilmot, R. L., Patton, J. C., and Bickham, J. W. (1993). Mitochondrial DNA variation in chinook *Oncorhynchus tshawytscha*) and chum salmon (*O. keta*) detected by restriction enzyme analysis of Polymerase Chain Reaction (PCR) products. *Can. J. Fish. Aquat. Sci.* 50, 708-715. doi: 10.1139/f93-081

Irwin, D. M., Kocher, T., and Wilson, A. C. (1991). Evolution of cytochrome b gene in mammals. *J. Mol. Evol.* 2, 13–34. doi: 10.1007/BF02515385

Kocher, T. D., Thomas, W. K., Meyer, A., **Edwards, S. V., Paabo, S., Villablanca, F. X., et al.** (1989). Dynamics of mitochondrial-DNA evolution in animals: amplification and sequencing with conserved primers. *Proc. Natl. Acad. Sci. U.S.A.* 86, 6196-6200. doi: 10.1073/pnas.86.16.6196

Meyer, A., Kocher, T. D., Basasibwaki P., and Wilson, A. C. (1990). Monophyletic origin of Victoria cichlid fish suggested by mitochondrial DNA sequences. *Nature* 347 (6293), 550-553. doi: 10.1038/347550a0
