# Supplementary material for: Genetic Pattern and Demographic History of Salminus brasiliensis: Population Expansion in the Pantanal Region during the Pleistocene
Source: Front Genet. 2018 Jan 17;9:1. doi: 10.3389/fgene.2018.00001 (PMC5776086; doi:10.3389/fgene.2018.00001)
Supplement: Supplementary file 3 [file Table_3.doc]

Table S3: Results of cluster analysis of the total (n=52) *Salminus brasiliensis* sample, using BAPS v6.0. Only the four best visited partitions are showed. For each possible number of distinct group (K) the log-likelihood and the probability are presented. Value in bold indicate the best K value.

| K | Log-likelihood | Probability |
| --- | --- | --- |
| **1** | **-1016.9731** | **1** |
| 2 | -1059.5519 | 0 |
| 3 | -1112.4062 | 0 |
| 4 | -1136.5774 | 0 |
