# Supplementary material for: Genetic Pattern and Demographic History of Salminus brasiliensis: Population Expansion in the Pantanal Region during the Pleistocene
Source: Front Genet. 2018 Jan 17;9:1. doi: 10.3389/fgene.2018.00001 (PMC5776086; doi:10.3389/fgene.2018.00001)
Supplement: Supplementary file 4 [file Table_4.doc]

Table S4: Priors distribution, demographic and mutation parameters used in the ABC analyses for each scenario. The unit of time is in generation. The generation time for *Salminus brasiliensis* is 2 years (de Godoy, 1975).

| Scenarios | Parameter | Interval (distribution) | Constraint on parameter |
| --- | --- | --- | --- |
| Constant population  (Scenario 1) | N_1_ | 10^5^ – 10^6^ (uniform) | - |
| Expansion population  (Scenario 2) | Na  N_2_  t2 | 10^3^ – 10^6^ (uniform)  10^3^ – 10^6^ (uniform)  2000 – 60000 (uniform) | Na<N_2_ |
| Decline population  (Scenario 3) | Na  N_3_  t2 | 10^3^ – 10^6^ (uniform)  10^3^ – 10^6^ (uniform)  2000 – 60000 (uniform) | Na>N_3_ |
| Old bottleneck  (Scenario 4) | Na  N_bot_  N_4_  t2  t1 | 10^3^ – 10^6^ (uniform)  10^2^ – 10^5^ (uniform)  10^3^ – 10^6^ (uniform)  2000 – 60000 (uniform)  1000 – 60000 (uniform) | t1<t2  Na>N_bot_  N_4_>N_bot_ |
| Old Expansion  (Scenario 5) | Na  N_exp_  N_5_  t2  t1 | 10^3^ – 10^6^ (uniform)  10^3^ – 10^6^ (uniform)  10^3^ – 10^6^ (uniform)  2000 – 60000 (uniform)  1000 – 60000 (uniform) | t1<t2  Na<N_exp_  N_5_<N_exp_ |
| All scenarios | µ | 10^-8^ – 10^-7^ (uniform)^1^ | - |

Na is the effective size of ancestral population; N_number_ is the effective size of present population in each scenario; N_bot_ is the effective population size during the bottleneck event; N_exp_ is the effective population size during the expansion event; t2 and t1 are the time in generation where happen a variation in population size; µ is the mutation rate per site per generation for each mitochondrial marker.

^1^ Cornuet, J. M. Ravigné, A. and Estoup, A. (2010). Inference on population history and model checking using DNA sequence and microsatellite data with the software DIYABC (v1.0). *BMC Bioinformatics* 11, 401. doi: 10.1186/1471-2105-11-401

Reference

de Godoy, M.P. (1975). *Peixes do Brasil, subordem Characoidei, Bacia do Rio Mogi Guassu*. Primeira edição. Editora Franciscana. Brazil. Vol II.
