# Supplementary material for: Genetic Pattern and Demographic History of Salminus brasiliensis: Population Expansion in the Pantanal Region during the Pleistocene
Source: Front Genet. 2018 Jan 17;9:1. doi: 10.3389/fgene.2018.00001 (PMC5776086; doi:10.3389/fgene.2018.00001)
Supplement: Supplementary file 5 [file Table_5.doc]

Table S5: Genetic diversity in different populations of *Salminus brasiliensis* from Upper Paraguay basin.

| River/  population | Sample size | CytB (710 bp) | | |  | Dloop (571 bp) | | |
| --- | --- | --- | --- | --- | --- | --- | --- | --- |
|  |  | Number of haplotype | Haplotype  diversity (Hd) | Nucleotide diversity (п) |  | Number of haplotype | Haplotype  diversity (Hd) | Nucleotide diversity (п) |
| Cuiabá | 10 | 8 | 0.952 | 0.00322 |  | 10 | 1 | 0.01611 |
| Formoso | 5 | 3 | 0.8 | 0.00225 |  | 5 | 1 | 0.01646 |
| Jaurú | 2 | 2 | 1 | 0.00282 |  | 2 | 1 | 0.02102 |
| Manso | 1 | 1 | - | - |  | 1 | - | - |
| Miranda | 6 | 6 | 1 | 0.00385 |  | 5 | 0.933 | 0.01074 |
| Paraguai | 12 | 7 | 0.879 | 0.00359 |  | 10 | 0.97 | 0.01486 |
| São Lourenço | 6 | 6 | 1 | 0.00554 |  | 6 | 1 | 0.01529 |
| Sepotuba | 5 | 4 | 0.9 | 0.00310 |  | 5 | 1 | 0.01226 |
| Taquari | 5 | 5 | 1 | 0.00451 |  | 4 | 0.9 | 0.01296 |
|  |  |  |  |  |  |  |  |  |
| All rivers | 52 | 21 | 0.928 | 0.00365 |  | 33 | 0.9751 | 0.01434 |
