# Supplementary material for: Genetic Pattern and Demographic History of Salminus brasiliensis: Population Expansion in the Pantanal Region during the Pleistocene
Source: Front Genet. 2018 Jan 17;9:1. doi: 10.3389/fgene.2018.00001 (PMC5776086; doi:10.3389/fgene.2018.00001)
Supplement: Supplementary file 6 [file Table_6.DOC]

Table S6: Bayesian posterior probabilities for each scenario estimated using DIYABC v2.1.0. The highest posterior probability is in bold.

| Scenarios | Posterior probability (95% Confidence Interval) |
| --- | --- |
| Scenario 1: Constant population | 0.0230 (0.0178 - 0.0281) |
| **Scenario 2: Old expansion** | **0.5466 (0.5341 - 0.5590)** |
| Scenario 3: Old decline | 0.0048 (0.0000 - 0.0099) |
| Scenario 4: Old bottleneck + expansion | 0.2589 (0.2466 - 0.2712) |
| Scenario 5: Old expansion + decline | 0.1668 (0.1582 - 0.1753) |
