# Supplementary figures and images for: Genetic Pattern and Demographic History of Salminus brasiliensis: Population Expansion in the Pantanal Region during the Pleistocene
Source: Front Genet. 2018 Jan 17;9:1. doi: 10.3389/fgene.2018.00001 (PMC5776086; doi:10.3389/fgene.2018.00001)

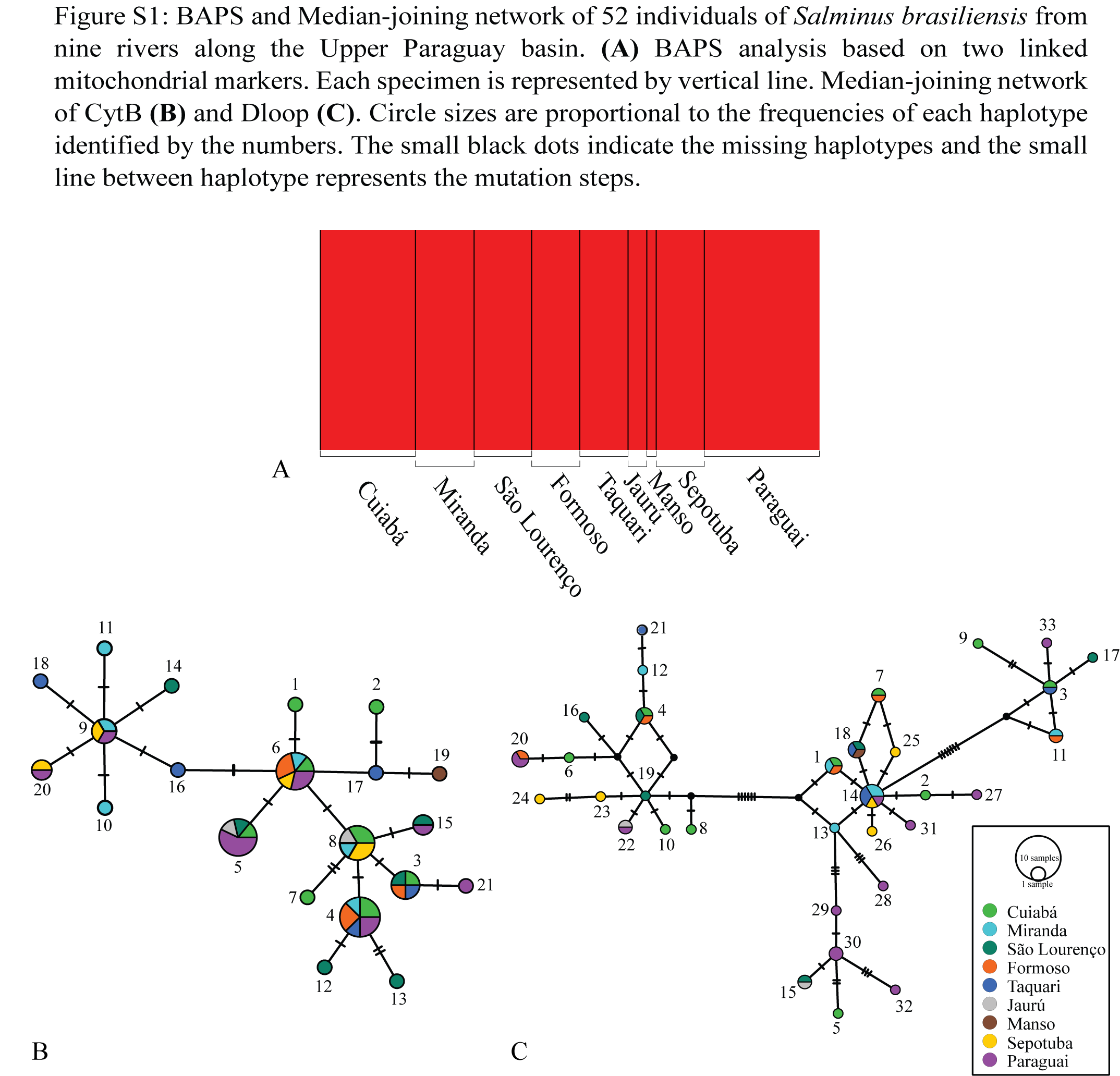

Supplement: Supplementary file 7 [file Image_1.tif]

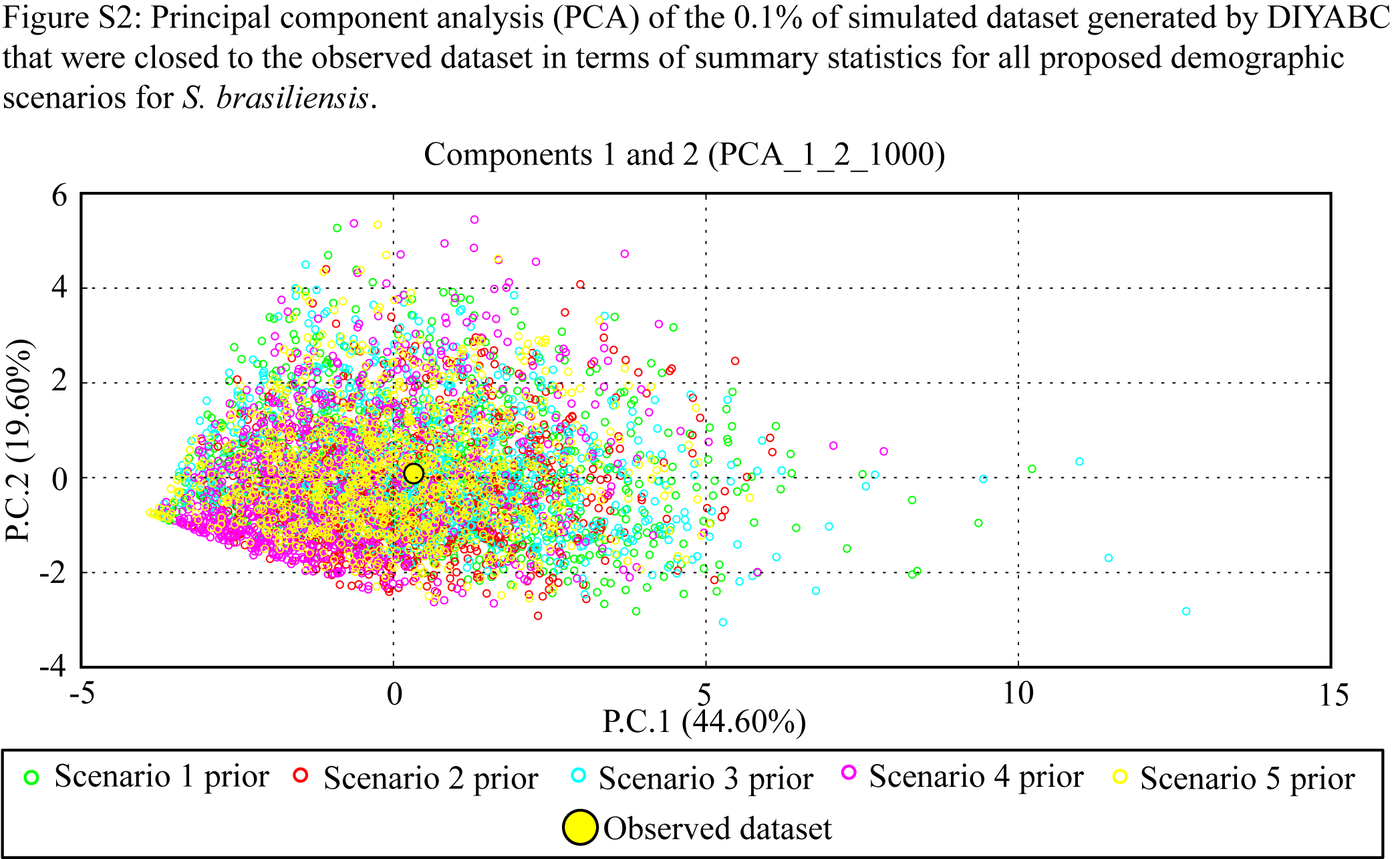

Supplement: Supplementary file 8 [file Image_2.TIF]

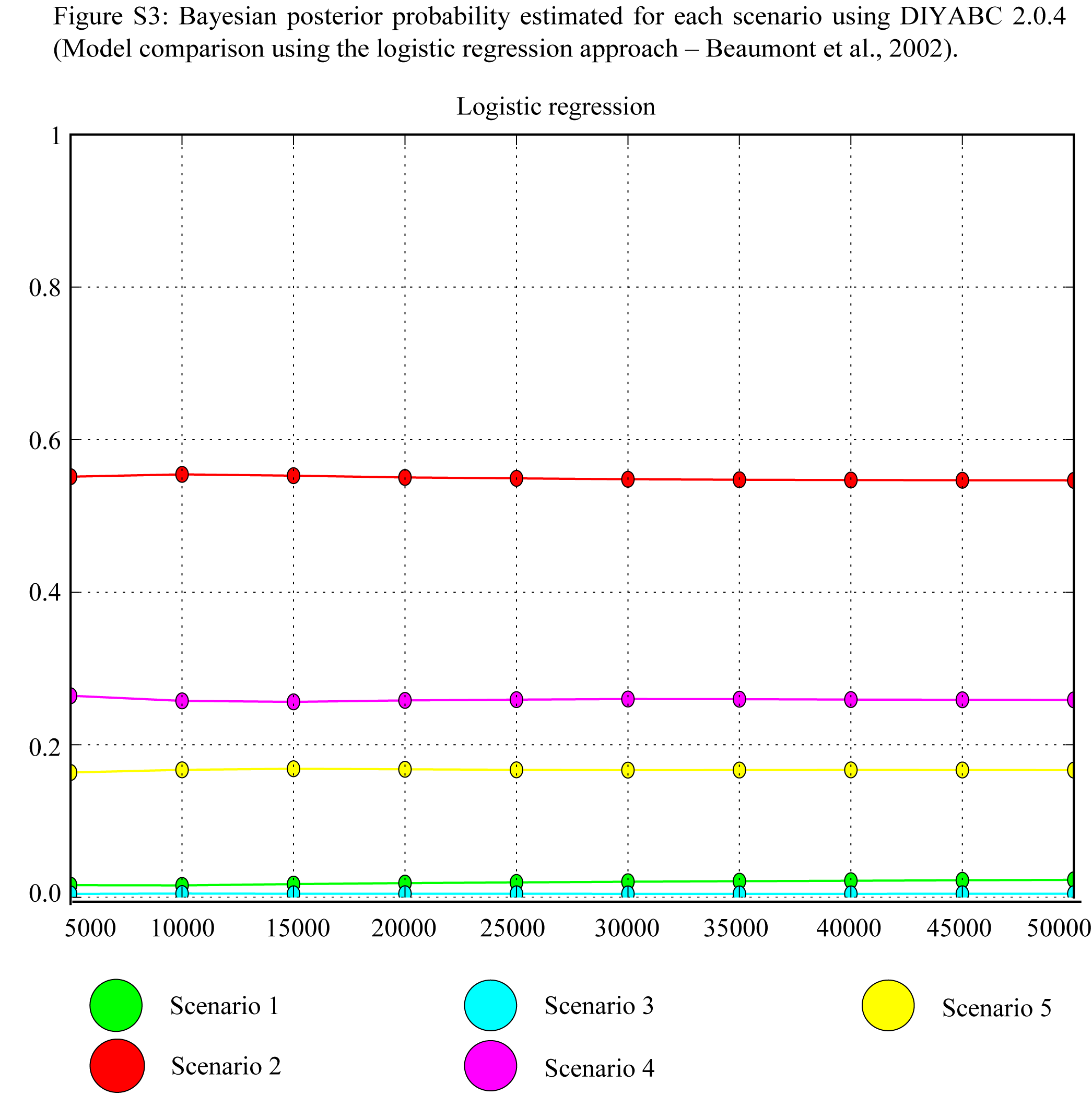

Supplement: Supplementary file 9 [file Image_3.tif]

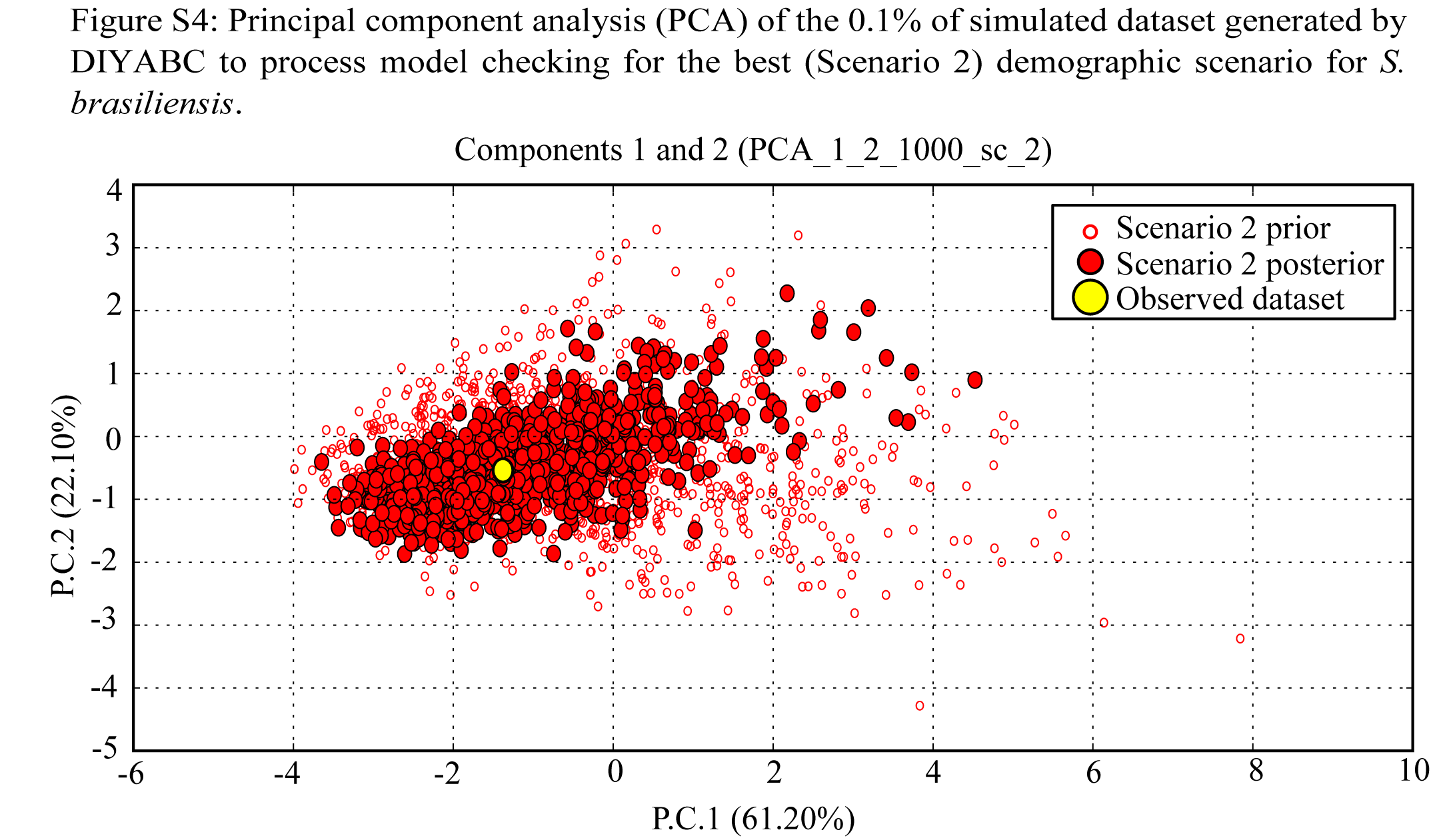

Supplement: Supplementary file 10 [file Image_4.TIF]
